# A role for consolidation in cross-modal category learning

**DOI:** 10.1016/j.neuropsychologia.2017.11.010

**Published:** 2018-01-08

**Authors:** Jennifer E. Ashton, Elizabeth Jefferies, M. Gareth Gaskell

**Affiliations:** Department of Psychology, University of York, Heslington, York YO10 5DD, UK

**Keywords:** Memory, Sleep, Consolidation, Categorization

## Abstract

The ability to categorize objects and events is a fundamental human skill that depends upon the representation of multimodal conceptual knowledge. This study investigated the acquisition and consolidation of categorical information that required participants to integrate information across visual and auditory dimensions. The impact of wake- and sleep-dependent consolidation was investigated using a paradigm in which training and testing were separated by a delay spanning either an evening of sleep or daytime wakefulness, with a paired-associate episodic memory task used as a measure of classic sleep-dependent consolidation. Participants displayed good evidence of category learning, but did not show any wake- or sleep-dependent changes in memory for category information immediately following the delay. This is in contrast to paired-associate learning, where a sleep-dependent benefit was observed in memory recall. To replicate real-world concept learning, in which knowledge is acquired across multiple distinct episodes, participants were given a second opportunity for category learning following the consolidation delay. Here we found an interaction between consolidation and learning; with greater improvements in category knowledge as a result of the second learning session for those participants who had a sleep-filled delay. These results suggest a role for sleep in the consolidation of recently acquired categorical knowledge; however this benefit does not emerge as an immediate benefit in memory recall, but by enhancing the effectiveness of future learning. This study therefore provides insights into the processes responsible for the formation and development of conceptual representations.

## Highlights

• Investigation into sleep- and wake-associated consolidation on declarative memory and cross-modal category learning.
• Declarative memory showed immediate sleep-associated improvements.
• Category knowledge remained stable across all consolidation delays, with no sleep-associated changes in performance.
• Sleep-associated consolidation did however increase the effectiveness of post-delay category learning.

## 1 Introduction

Conceptual knowledge refers to the information we possess that enables us to bring meaning to the words, objects and events we encounter daily (Lambon Ralph et al., 2010, Lambon Ralph et al., 2016). This information is essential for communication and cognition and draws on abstract representations that describe the categorical and functional relationships between items (Kintsch and Walter, 1988). The development of conceptual knowledge is thought to require the integration of information across different sensory modalities (e.g. vision and sound) and multiple learning episodes, giving rise to higher-order similarity structures that take into account all available sources of information (Lambon Ralph et al., 2016, Patterson et al., 2007). For any given concept, cross-modality integration is important, as similarity in one modality may not be sufficient to extract appropriate conceptual relationships. For example; pears and light bulbs are similar in shape but are not related in meaning. Studies investigating perceptual category learning provide successful demonstrations of feature integration to order to develop conceptual representations (Ashby and Ell, 2001, Ashby et al., 2003, Ashby and Valentin, 2005, Ashby and Casale, 2003). However, little research has focused upon the acquisition of cross-modal representations and in particular their development across time (Maddox et al., 2006, Maddox et al., 2009, Hennies et al., 2014).

**Fig. 1:**
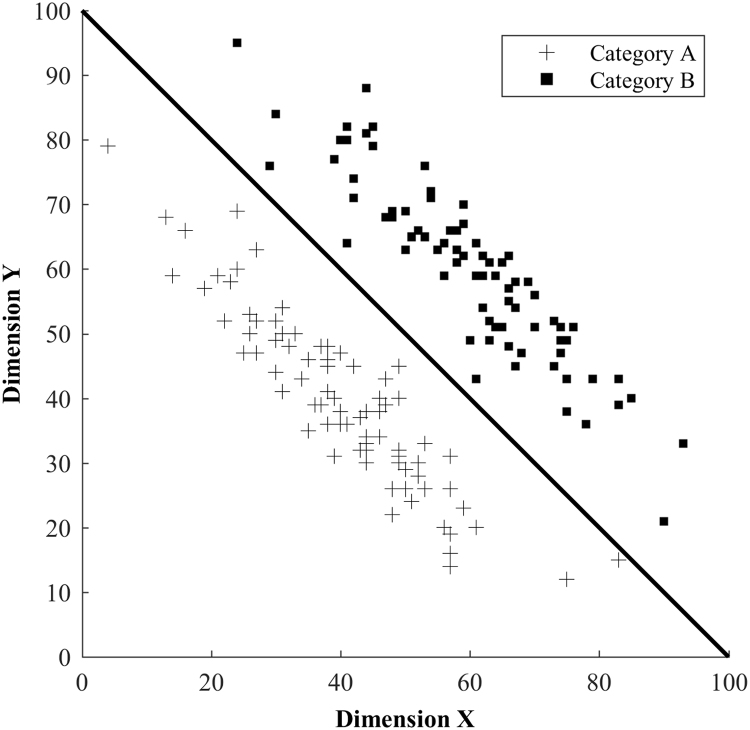
An information-integration category structure. The stimuli are depicted within an abstract space, with each dimension having 100 levels. Both dimensions carry useful category information; but successful (optimal) categorization requires integration.

To study the acquisition of cross-modal category representations, it is necessary to create arbitrary ‘artificial’ categories. The categorization literature provides a useful paradigm for creating such stimuli and allows the underlying structure of the categories to be experimentally manipulated in order to promote integration across multiple features or dimensions. Categories that require the integration of two (or more) stimulus dimensions are referred to as information-integration category structures (an example is presented in Fig. 1). When presented with stimuli from this type of structure, information about category identity is available in both dimensions; however neither dimension alone is sufficient to make precise categorizations. For optimal categorization, information from both dimensions needs to be integrated in order to determine the category boundary (the bold line in Fig. 1 shows the optimal category boundary). Through feedback-driven exposure to category exemplars, participants are able to acquire knowledge of information-integration category structures and show high levels of categorization accuracy (Ashby and Maddox, 2005, Ashby and Maddox, 2011).

Most studies within the categorization literature have focused on two-dimensional category structures within a single (visual) domain (e.g. Gabor patches – sinusoidal gratings that vary on the dimensions of orientation and frequency) overlooking the cross-modal nature of much conceptual knowledge. However, information-integration category structures can be created using cross-modal stimuli; Maddox et al. (2006) used visual-auditory stimuli dimensions, and subsequent work has shown high levels of categorization when the category structure is manipulated such that the categories overlap (Smith et al., 2014). In accordance with these findings and to capture the cross-modal nature of conceptual knowledge, the current study utilised a cross-modal (visual-auditory) information-integration categorization paradigm to study the development of category knowledge across time.

Research investigating the development of memory across time has typically focused upon episodic declarative memory, which requires rapid learning at a specific point in time. However, conceptual information is extracted from features present across multiple spatially and temporally distinct episodes (Rogers and McClelland, 2004). Given the gradual emergence of conceptual knowledge, it is therefore important to consider (i) the influence of consolidation processes that may occur in between learning episodes and (ii) the effects of prior learning on the information that can be extracted from new experiences.

There has been a large amount of research into memory consolidation; the processes that serve to maintain, strengthen and modify memories. These processes may occur across both wake and sleep; however tasks that assess episodic declarative memory suggest a specific role for sleep in memory consolidation (Diekelmann et al., 2009). One task that reliably demonstrates sleep-dependent consolidation benefits is paired-associate learning, in which participants are required to learn lists of associated word-pairs. Memory for the learned pairs is usually assessed using cued-recall procedures, which follows a post-learning delay that is manipulated to contain either sleep or wakefulness. Consistently, studies report better memory retention after a delay containing sleep (compared to wake) suggesting a role for sleep-dependent consolidation in long-term memory retention (Jenkins and Dallenbach, 1924, Plihal and Born, 1997, Tucker et al., 2006, Diekelmann et al., 2009).

It was originally hypothesised that sleep benefits memory by offering passive protection from interference and forgetting (Ellenbogen et al., 2006). However, there is now strong evidence to suggest that sleep plays an active role in consolidation by promoting systems-level memory transfer (Diekelmann and Born, 2010). The active systems consolidation hypothesis suggests that during sleep, newly encoded information is integrated within long-term memory networks and is reorganised to enable the extraction of invariant features (Born and Wilhelm, 2012). Strong support for the specific role of sleep has been provided by numerous studies which show a correlation between the change across a sleep delay and sleep physiology, specifically slow-wave sleep (SWS) (for a review see Rasch and Born, 2013). Causal evidence is provided by studies which have re-exposed participants to encoding associated cues (e.g. odours or auditory cues) during SWS – which leads to enhanced memory performance, highlighting a role for memory reactivation as a possible mechanism of sleep-associated consolidation (Rasch et al., 2007, Rudoy et al., 2009, Rasch and Born, 2013). Consolidation during sleep is therefore thought to not only strengthen individual representations, but also to facilitate the extraction of shared and systematic features from the environment – a potentially critical mechanism for the development of concept or categorical memory representations. Sleep-dependent consolidation beyond isolated episodic memories has received much less attention; however there is evidence to suggest that sleep plays a role in the extraction of regularities (Lau et al., 2011). Ellenbogen et al. (2007) used a transitive inference paradigm to examine the role of wake- and sleep-dependent consolidation on the extraction of an implicit hierarchical structure. Participants learned arbitrary “premise pairs” (e.g. A > B, B > C, C > D etc.) followed by a wake- or sleep-filled post-learning delay. Participants were then tested on their memory for the trained pairs (e.g. A > B) and their knowledge of the untrained hierarchy (e.g. B > D). The two groups showed comparable memory for trained items; however the sleep group outperformed the wake participants when knowledge of the more distant untrained hierarchy was assessed, suggesting sleep had facilitated extraction of the underlying hierarchical information (Ellenbogen et al., 2007).

A sleep-dependent benefit for the extraction of regularities is not however consistently reported. In a declarative language learning task, Mirković and Gaskell (2016) report sleep-dependent benefits for arbitrary vocabulary knowledge, but fail to find differences between wake and sleep groups when assessing knowledge for systematic aspects of the trained language (i.e. grammatical regularities). It is these systematic aspects of learning that are thought to contribute to conceptual memory; however few studies take into account the real-world nature of conceptual learning which develops across distinct episodes. Evidence from animals (Tse et al., 2007), humans (van Kesteren et al., 2013) and computational models (McClelland, 2013) suggests that new learning is facilitated by prior schematic knowledge, with accelerated integration when new and existing information are consistent (McClelland, 2013). The acquisition of conceptual information across time may therefore rely heavily on an interaction between consolidation processes and subsequent learning episodes. A single post-delay test, the typical procedure used in consolidation research, may therefore fail to capture the true impact of consolidation on the development of conceptual knowledge across time. In an attempt to replicate realistic category learning, and to capture potential interactions between consolidation and learning mechanisms, this study included a second learning opportunity following the consolidation delay.

To our knowledge, two studies have used the information-integration categorization task described above to study the development of category knowledge across time. Maddox et al. (2009) examined the influence of sleep deprivation on information-integration category learning. They provided category training in two sessions separated by 24-h during which participants were kept awake or were able to maintain their usual wake-sleep cycle. Maddox et al. reported poorer performance for participants who remained awake between sessions, however, due to the sleep deprivation paradigm, this study cannot separate the effects of sleep-based consolidation from those of fatigue.

A second study reports an offline consolidation benefit in category learning when comparing a delay of 24-h with 15-min (Hennies et al., 2014). Unlike immediate post-delay consolidation effects which are reported in studies assessing episodic declarative memory, the benefit in this study emerged only after further training following the delay; suggesting a subtle benefit of consolidation which increased the effectiveness of post-delay learning. Hennies et al. (2014) went on to compare the effects of sleep and wake separately by using a 12-h delay that spanned either a night of sleep or a day of wakefulness; they found a specific consolidation benefit for the wake, but not the sleep, delay condition. This result contrasts with those typically observed within the consolidation literature and suggests that categorization may not benefit from sleep-based consolidation in the same way as declarative memory. However, Hennies et al. (2014) made a number modification to the categorization paradigm. These changes made the information-integration structure predictive of category membership, but secondary to categorization – which was based on a one-dimensional visual rule that was provided to participants. This is likely to have had a large impact on learning in the task, given that participants were not required to use the category structure to achieve accurate categorization. Furthermore, in contrast to the typical measurement of accuracy that is used in categorization studies, their measurement of integration was based upon changes in reaction time, making it difficult to compare their results with the existing categorization literature. In the current study, we wanted to assess the role of wake and sleep based consolidation using the traditional, and unmodified, information-integration category learning structure.

Thus, while the role of sleep-dependent consolidation in the development of episodic declarative memory is relatively well-established, the contribution of consolidation in the development of conceptual memory has not been widely investigated. It is unknown whether the behavioural consequence of sleep-dependent consolidation is consistent across memory types, or indeed whether sleep- or wake-dependent mechanisms have a specific role to play in the consolidation of conceptual memory. The potential influence of such a mechanism on the stabilization of previously encoded information and the impact on subsequent learning has yet to be established.

Accordingly, the current study investigated the role of consolidation on both traditional paired-associate declarative memory and conceptual categorization in a cross-modal information-integration paradigm (Ashby and Gott, 1988). Basic two-dimensional cross-modal (auditory-visual) stimuli were created and participants were expected to demonstrate sensory integration in order to form cross-modal categorical representations. By employing a 15-min and 12-h sleep or wake delay between two sessions of learning, we assessed independent contributions of time and of wake- and sleep-dependent consolidation on (i) the retention of previously-encoded episodic and categorical representations, and (ii) the capacity to further develop category knowledge after consolidation. The effects of sleep were then replicated in a second sample with concurrent polysomnography recordings although for ease of exposition all groups are presented in the same analysis.

## 2 Methods

### 2.1 Participants

Participants were 95 undergraduate students recruited from the University of York in fulfilment of course credit or for payment. Participants reported normal or corrected-to-normal vision and hearing and were assigned to one of four experimental conditions: a 12-h wake group (n = 23, mean age: 20.52, S.D. ± 3.54, 17 female), a 12-h sleep group (n = 22, mean age: 20.05, S.D. ± 1.32, 19 female), a PSG-monitored overnight sleep group (n = 23, mean age: 20.87, S.D. ± 2.49, 16 female) or a 15-min delay group (n = 27, mean age: 20.67, S.D. ± 3.54, 21 female). Participants in the overnight PSG-monitored sleep group were required to be free from psychoactive drugs, including alcohol and caffeine, and to refrain from daytime napping for 24 h preceding and throughout the study period.

### 2.2 Study overview

All participants were tested on a measure of declarative episodic memory (paired-associate learning) and a conceptual category learning task. Participants completed two sessions of the study; to assess paired-associate memory a typical consolidation paradigm was utilised where participants completed encoding and immediate cued-recall in session 1, followed by a delayed cued-recall test in session 2. Category training followed a similar procedure, however following the delayed test in session 2, participants completed a second round of training and a final test before completing a number of categorization follow-up tasks. The two sessions were separated by a delay of varying lengths (15-min vs. 12-h) that were manipulated to separately assess the contribution of wake- and sleep-dependent consolidation.

### 2.3 Experimental tasks

#### 2.3.1 Paired-associate learning

##### 2.3.1.1 Paired-associate stimuli

80 words were selected from an adapted version of The University of South Florida (USF) word association, rhyme, and word fragment norms (Nelson et al., 2004) to create 40 semantically unrelated cue and target word pairs (e.g. owl – frame). Both the cue and target words were singular, had high USF concreteness ratings (cues = 5.90 ± .61; targets = 5.85 ± .41,*t*(39) = .39; *p* = .696) and were matched for frequency (cues = 35.10 ± 41.09; targets = 40.73 ± 55.26, *t*(39) = −4.71; *p* =.640), word length (cues = 5.18 ± 1.34; targets = 5.15 ± 1.05, *t*(39) = .09; *p* =.933) and number of syllables (cues = 1.45 ± .68; targets = 1.55 ± .60, *t*(39) = −.73; *p* = .472). There were no pre-existing forward- or backward-association relationships between any of the words, reducing the likelihood of erroneous associations between words in separate pairs.

##### 2.3.1.2 Paired-associate encoding

Participants were presented with each word pair for 5000 ms and were instructed to memorize the two words as a pair for a future memory test. To help memorize the word pairs participants were instructed to use visual imagery.

##### 2.3.1.3 Paired-associate immediate recall

To test their memory immediately after encoding, participants were presented with the cue from each pair (i.e. the first word of the pair) and given 10 s to recall the target word (i.e. the second word of the pair). Participants made their responses by typing the target word into the computer; they were instructed to use the backspace if they made a mistake and pressed the enter key to submit their response. Participants received immediate feedback following each response (3500 ms), and on incorrect trials the correct cue and target was re-presented and participants were instructed to try to re-learn that word-pair. Cued-recall with feedback offers the opportunity for extra learning for incorrectly recalled pairs. As a result, it is expected that memory accuracy will increase between this and future memory tests. This immediate recall procedure was repeated until participants correctly recalled a minimum of 60% of the word pairs, or until they had completed the recall procedure a maximum of three times. This criterion was set to try and maintain a similar level of performance across participants, without large differences in the number exposures to the stimuli.

##### 2.3.1.4 Paired-associate delayed recall

Delayed recall followed the same procedure as immediate recall; however participants did not receive feedback on their performance and completed the task just once.

#### 2.3.2 Categorization task

##### 2.3.2.1 Category stimuli

**Fig. 2:**
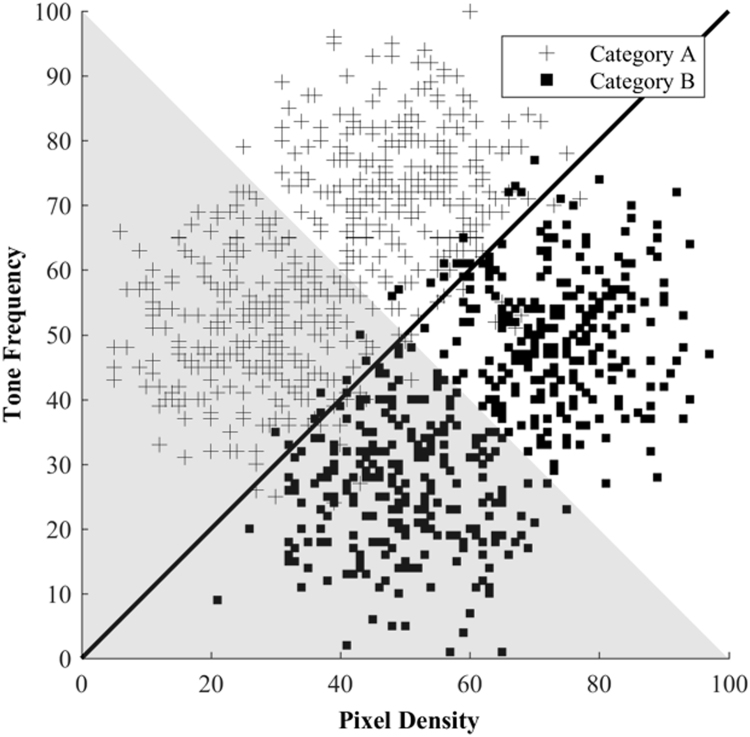
The information-integration category structure normalised to a 100 point scale. The solid line denotes the optimal linear decision boundary; the corsairs and squares represent Category A and Category B respectively. Items that fall within the shaded region were presented on the left hand side of the screen and those in the non-shaded region presented on the right hand side of the screen.

All stimuli were generated using MATLAB (PsychToolBox). Category exemplars were two-dimensional conjoint visual-auditory stimuli based on Smith et al. (2014). The visual dimension was a 150 × 150 pixel unframed box containing randomly placed yellow pixels, presented on a black background. There were one hundred-and-one levels of pixel density with the number of yellow pixels at each level defined by *pixels = round(850 × 1.0181*^*level*^*)*. Pixel density therefore varied from 850 lit pixels (level 0), to 5061 lit pixels (level 100) out of a total of 22,500. The auditory dimension was a pure tone that varied in frequency (Hz), defined by *frequency = 220 × 2*^*(level/120)*.^ For levels 0 and 100 the pitches were 220 Hz and 392 Hz respectively. Stimuli were presented on the right- or left-hand side of the screen. The placement of each stimulus was determined by its position within the stimulus space (see Fig. 2); a boundary line orthogonal to the category boundary separated the stimuli, with trials on one side of the boundary presented on the left hand side of the screen during training (the shaded area in Fig. 2) and trials on the other side presented on the right hand side of the screen (the non-shaded area in Fig. 2). Although systematic, screen location did not provide any information about category identity and was therefore considered task-irrelevant.

##### 2.3.2.2 Category structure

Category exemplars were created using Ashby and Gott's (1988) randomization technique. Categories were defined by bivariate distributions along the two stimulus dimensions following the information-integration condition of Filoteo et al. (2010) (see Table 1). Each stimulus was created by drawing a random sample (x, y) from the stimulus space. Stimuli sets were created for each individual, with each set normalised to match the overall category distribution before being transformed into concrete visual and auditory stimuli using the formulae above. This normalisation ensured that each participant had the same statistical information, despite receiving their own unique set of individual exemplars. Maximum accuracy using the optimal linear boundary as shown in Fig. 2 would be 95% as there is a 5% category overlap.

**Table 1:** Category distribution parameters (mean (µ) and standard deviation (σ)) for the pixel density (x) and tone frequency (y) dimensions in the information integration category structure.

	Category Parameters			
Category	µ_x_	µ_y_	σ_x_	σ_y_
A	26.67	50.00	10	10
A	50.00	73.33	10	10
B	50.00	26.67	10	10
B	73.33	50.00	10	10

##### 2.3.2.3 Category learning trials

**Fig. 3:**
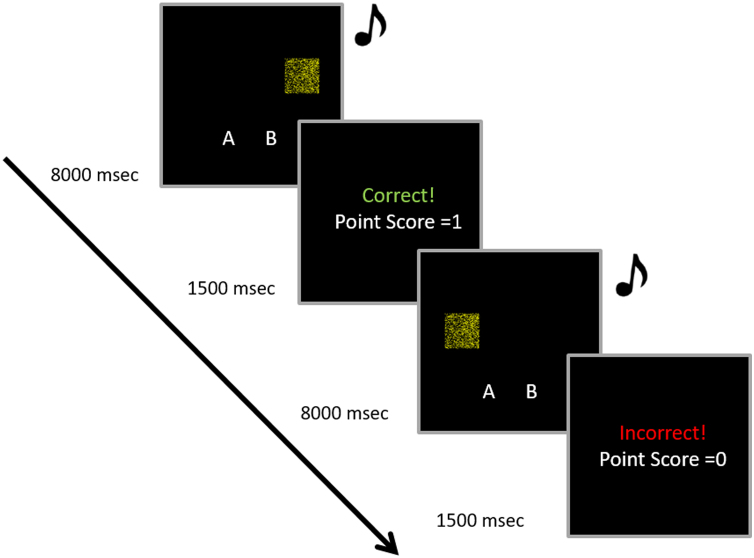
Sequence of events for two trials in the categorization task.

Participants completed two blocks of sixty trials in each learning session, (with 60 Category A and 60 Category B trials presented in a randomised order). On each trial, one conjoint visual-auditory category exemplar was presented. The response icons ‘A’ and ‘B’ were presented in the lower left- and right- hand side of the screen, and participants were asked to categorize each stimulus by pressing the ‘A’ or ‘B’ keyboard keys. The stimuli were presented for a maximum of 8 s and terminated immediately following a response; if no response was given with the 8 s the trial ended and this was scored as incorrect. Participants received immediate feedback following each response, with the word “Correct!” or “Incorrect!” presented in the centre of the screen. To encourage good performance and to engage participants throughout the task a points system was used such that points were added or deducted from a running total following each response. A monetary reward was offered for the highest performing participant. A detailed example of two trials from the category learning task is presented in Fig. 3.

##### 2.3.2.4 Instructions

Participants were told that each trial of the categorization task contained a pixel box and an auditory tone, with the chance of each trial belonging to category A or B being equal. They were instructed to categorize each trial by pressing the “A” or “B” keyboard key and that they would need to guess at first, but with practise they would be able to categorize the stimuli accurately. Participants were instructed to focus on the density of the pixels and the pitch of the tone to make their decisions; they were informed that the pixel box would be located on the left or right hand side of the screen, but that this was not important for making their categorization decisions. Participants were encouraged to focus on being as accurate as possible during learning.

#### 2.3.3 Categorization follow-up tasks

Follow-up tasks aimed to assess participants’ knowledge of the category structure, as learned in the categorization task. The stimuli used in these tasks were the same as described above.

##### 2.3.3.1 Categorization test

**Fig. 4:**
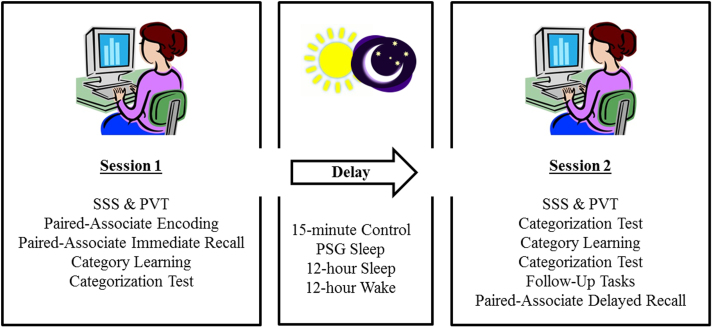
Experimental procedure. Participants completed both sessions and were allocated to one of four delay conditions. (SSS – Stanford Sleepiness Scale, PVT – Psychomotor Vigilance Task).

The categorization test included 60 trials which followed a similar procedure to categorization learning; however participants did not receive feedback on their performance. A fixation-cross of 1500 ms was presented before the onset of the each trial and participants were instructed to respond both as accurately and as quickly as possible, using the knowledge they had gained during learning to guide their decisions. Participants performed the categorization test three times; immediately following learning in session one, straight after the delay in session two and finally after the second round of category training in session two (see Fig. 4).

##### 2.3.3.2 Two-alternative forced choice (2AFC) task

Participants completed a 2AFC task to assess their ability to identify category exemplars. On each trial participants were presented with a ‘target Category’ (either A or B) in the centre of the screen. The task was divided such that in half of the trials they were presented with a single auditory tone, and two pixel boxes (pixel trials) while in the other half of trials they were presented with one pixel box and two auditory tones (tone trials). In both trial types, stimuli could be combined to make legitimate category A or B items. The participants' task was to select the stimuli they thought combined to create an exemplar of the target category. For example, on ‘pixel trials’ participants had to select (from the two pixel boxes) the one they thought combined with the auditory tone to match the target category. Participants completed 80 trials in total (40 pixel trials, 40 tone trials) and were instructed to respond as accurately as possible; a fixation cross (1000 ms) preceded the onset of each trial.

##### 2.3.3.3 Recall task

Participants completed a recall task to assess their ability to generate category exemplars. On each trial participants were presented with a scale which represented the normalised level of either the density of a pixel box or the frequency of a tone (ranging from level −25 to 125). They were also presented with a ‘target category’ (either A or B) in the centre of the screen, along with a fixed stimulus from one dimension (e.g. a pixel box). Their task was to change the scale representing the non-presented dimension (e.g. the frequency of the tone) to match the target category. Participants used the mouse to click their chosen position on the scale and were able to change position an unlimited amount of times. In half of the trials the fixed dimension was the pixel box, while in the other half of trials the tone was fixed. Participants were instructed to be as accurate as possible. Each trial was preceded by a fixation cross presented for 2000 ms and participants completed 60 trials in total (30 of each type).

##### 2.3.3.4 Location task

The location task was used to assess participants’ knowledge of the task-irrelevant location dimension. This was considered to be task irrelevant as screen location did not provide any cues to category membership. We included this manipulation to assess whether participants were sensitive to information that was not relevant for successful categorization and if knowledge of this information developed differently across delays containing sleep or wake. On each trial they were provided with a conjoint visual-auditory stimulus and its category in the centre of the screen. They had to indicate whether they believed the stimulus belonged on the left or right hand side of the screen. Each trial was preceded by a fixation cross for 1000 ms and participants were instructed to respond as accurately and as quickly as possible. They completed 60 trials in total.

#### 2.3.4 Psychomotor vigilance task (PVT)

The PVT is a sustained-attention, reaction-timed task that measures the speed with which participants respond to visual stimuli. The PVT task was obtained from http://bhsai.org/downloads/pc-pvt/ (Khitrov et al., 2014). During the task, participants were presented with a blank black screen; at random intervals, a millisecond counter began to scroll, and participants had to left click the mouse to stop the counter as quickly as possible. After clicking, the counter displayed the achieved reaction time for 1000 ms, providing the subject with feedback on performance. Inter-stimulus intervals were distributed randomly from 2 to 10 s, and the task lasted for a total of 3 min.

### 2.4 Procedure

The experiment consisted of two experimental sessions separated by a delay of varying lengths across the four conditions. The two 12-h delay groups spanned either daytime wakefulness, in which participants continued with their usual daytime activities, or an evening of sleep, where participants returned home to sleep. For these two groups Session 1 began at 8.30 a.m. and 8.30 p.m. respectively with Session 2 being completed exactly 12-h later. Participants in the overnight PSG group were required to arrive at the lab at 8.30 p.m. and completed the experimental tasks after PSG set-up (9.45 p.m. ± 30 min). These participants remained in the lab to sleep and were awoken from sleep at approximately 7.30 a.m.; they completed Session 2 tasks at 8.30 a.m. Participants in the 15-min delay group completed Session 1 between 9.00 a.m. and 12.00 p.m. These participants were instructed to take a 15-min break and were encouraged to leave the testing lab in order to avoid fatigue before completing Session 2.

A schematic illustration of the experimental procedure is shown in Fig. 4. Both sessions began with completion of the Stanford Sleepiness Scale (SSS) (Hoddes et al., 1973) followed by the PVT to obtain measures of sleepiness, alertness and vigilance. In Session 1, participants completed paired-associate encoding and immediate cued-recall recall, followed by category learning and the first categorization test (Session 1 ~ 45 min). Session 2 tasks involved a second categorization test, a further session of category learning and a final categorization test. Participants then completed the categorization follow-up tasks and finally paired-associate delayed recall (Session 2 ~ 1 h).

### 2.5 Sleep recording with polysomnography (PSG)

For participants in the overnight PSG group, an Embla N7000 PSG system with RemLogic version 3.4 software was used to monitor sleep. After the scalp was cleaned with NuPrep exfoliating agent (Weave and Company), gold plated electrodes were attached using EC2 electrode cream (Grass Technologies). EEG scalp electrodes were attached according to the international 10–20 system at six standardised locations: central (C3 and C4), occipital (O1 and O2) and frontal (F3 and F4), and each was referenced to an electrode on the contralateral mastoid (A1 or A2). Left and right electrooculography electrodes were attached, as were electromyography electrodes at the mentalis and submentalis bilaterally, with a ground electrode attached to the forehead. Each electrode had a connection impedance of < 5 kΩ and all signals were digitally sampled at 200 Hz.

## 3 Results

Data were analysed in SPSS 23. All effects that reached a significance level of *p* < .1 are reported, with effects where *p* < .05 considered significant. Bonferroni-corrected *t*-tests were used to evaluate main effects for factors with more than two levels.

### 3.1 Stanford sleepiness scale and psychomotor vigilance task

Alertness measures were taken using the SSS (ratings of sleepiness) and performance on the PVT, focusing upon measures of reaction time (RT) and attentional lapses (RT > 500 ms, data is presented in Table 2). Each measure was analysed using analysis of variance (ANOVA) with the between-subjects variable Group (15-min, PSG, 12-h wake, 12-h sleep) and repeated-measures variable Session (Session 1, Session 2). There were no differences in the levels of rated sleepiness across groups (F(3, 90) = 2.36, *p* = .077), however there was a main effect of session, with participants rating themselves as sleepier in session one when compared to session two (F(1, 90) = 9.25, *p* = .003); there was no interaction between these factors (*p* > .69). No differences were observed when measuring alertness by mean RT (Group; F(1, 89) = 0.90, *p* = .443, Session; F(1, 89) = 0.001, *p* = .980) or the number of lapses in the PVT (Group; F(1, 89) = 0.39, *p* = .758, Session; F(1, 89) = 0.25, *p* = .620). This suggests that general levels of alertness cannot account for any effects of Group in the experimental tasks.

**Table 2:** Stanford Sleepiness Scale (SSS) and Psychomotor Vigilance Task (PVT) scores for each group in Session 1 and Session 2. SSS ratings are marked on a 7-point scale with a score of 1 representing most alert; mean scores are presented. PVT scores represent mean reaction time (RT) in ms and the mean number of lapses in attention (RT > 500 ms). Standard error of the mean is presented in brackets.

	Session 1			Session 2		
	SSS	PVT RT	PVT Lapse	SSS	PVT RT	PVT Lapse
15-min	2.73 (0.16)	254.75 (4.91)	0.08 (0.05)	2.23 (0.14)	272.96 (9.05)	0.65 (0.36)
PSG	3.17 (0.17)	279.25 (6.18)	0.17 (0.08)	2.52 (0.15)	275.51 (6.71)	0.52 (0.23)
12 h – Sleep	3.18 (0.20)	278.47 (9.83)	0.48 (0.19)	2.81 (0.23)	268.02 (6.16)	0.24 (0.10)
12 h – Wake	2.70 (0.23)	274.67 (7.42)	0.74 (0.27)	2.47 (0.21)	270.27 (5.77)	0.35 (0.12)

### 3.2 Paired-associate learning

Analysis of paired-associate memory focused upon accuracy in the final recall attempt from the immediate test (if participants were required to repeat the test to meet the 60% recall criterion) and delayed cued-recall. Two participants were removed from the analysis due to computer failures during delayed recall (both from the 15-min delay condition). To examine changes in performance across the delay, an analysis of covariance (ANCOVA) was performed on delayed recall with the variable Group (15-min, PSG, 12-h wake, 12-h sleep) and covariate immediate cued recall (see Table 3). The ANCOVA revealed a significant effect of Group (F(3, 93) = 10.02, *p* < .001, η^2^ = 0.26). Post-hoc Bonferroni-corrected pairwise comparisons showed that this effect was driven by a smaller proportion of correctly recalled items in the 12-h wake group compared to all other conditions (15-min delay *p* = .001, 12-h sleep *p* < .001, PSG overnight group *p* < .001). Therefore, in this assessment of episodic declarative memory, we observe a sleep-associated benefit for delayed cued-recall.

**Table 3:** Accuracy in the immediate paired associated cued-recall test (data taken from the final recall attempt, mean proportion correct presented) and delayed cued-recall (covariate adjusted means are presented with the covariate immediate recall). Standard error of the mean is presented in brackets.

	Paired-Associate Recall	
	Immediate Test	Delayed Test
15-min	0.73 (0.03)	0.84 (0.16)
PSG	0.71 (0.03)	0.86 (0.16)
12 h – Sleep	0.71 (0.02)	0.86 (0.17)
12 h – Wake	0.78 (0.03)	0.75 (0.16)

### 3.3 Category learning

#### 3.3.1 Categorization – session 1

The rate of category learning in Session 1 was assessed by comparing the number of correctly categorized trials in the two blocks of training. Performance is presented in Table 4 and was analysed using an ANOVA with the within-subjects variable Block (Block 1, Block 2) and between-subjects variable Group (15-min, PSG, 12-h wake, 12-h sleep). A main effect of Block was observed (F(1, 91) = 20.93, *p* < .001, η^2^ = 0.19), demonstrating improvements in categorization across training. There were no Group differences (F(3, 91), 0.44, *p* = .727) and no interaction between the variables (F(3, 91) = 0.96, *p* = .418).

**Table 4:** Performance in the categorization learning task and tests. Session 1 scores represent the mean proportion of correctly categorized trials. Session 2 scores show covariate adjusted means (as evaluated with the covariate Test 1). Standard error of the mean is presented in brackets.

	Session 1			Session 2			
	Learning		Test 1	Test 2	Leaning		Test 3
	Block 1	Block 2			Block 1	Block 2	
15-min	0.66 (0.02)	0.70 (0.02)	0.71 (0.02)	0.73 (0.01)	0.73 (0.02)	0.77 (0.02)	0.74 (0.02)
PSG	0.64 (0.02)	0.69 (0.02)	0.68 (0.03)	0.74 (0.02)	0.76 (0.02)	0.77 (0.02)	0.77 (0.02)
12 h – Sleep	0.65 (0.02)	0.72 (0.02)	0.76 (0.02)	0.71 (0.02)	0.74 (0.02)	0.80 (0.02)	0.77 (0.02)
12 h – Wake	0.66 (0.02)	0.69 (0.02)	0.75 (0.02)	0.71 (0.02)	0.73 (0.02)	0.73 (0.02)	0.69 (0.02)

The first categorization test provides a measure of Session 1 category learning. All groups performed above chance level, as determined by one-sample *t*-tests with chance level performance as 0.5 (*p* < .001 for all groups). Data is presented in Table 4 (Test1), a between-subjects ANOVA with the variable Group was non-significant (F (3, 91) = 1.85, *p* = .143). There was however some variation in condition means and so performance at this time-point was used as a covariate in subsequent analyses.

#### 3.3.2 Categorization – session 2

**Fig. 5:**
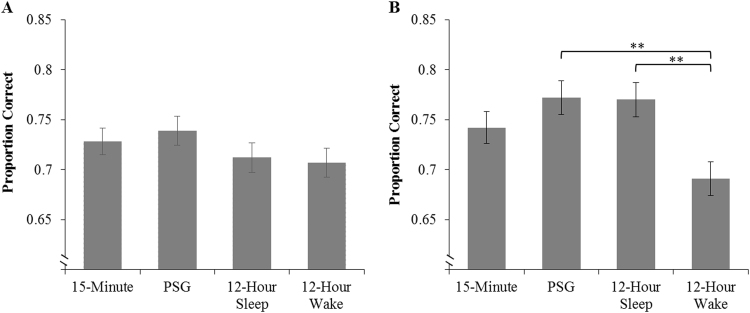
The proportion of correctly categorized trials during Test 2 (A) and Test 3 (B). Residual proportions are presented as evaluated with the covariate Test 1. Error bars represent SEM. (** represents *p* < .01).

Category knowledge was re-assessed with a test at the beginning of Session 2 to measure the retention of category knowledge across the delay. Again all groups performed above chance level (0.5) when tested with one-sample *t*-tests (*p* < .001 for all groups). Performance in this test (see Fig. 5a) was assessed using an ANCOVA with the variable Group (15-min, PSG, 12-h wake, 12-h sleep) and covariate Test 1. A non-significant effect of Group suggests that all groups were performing at a similar level (F(3, 90) = 1.00, *p* = .397). There was no evidence for immediate consolidation effects on the retention and retrieval of categorical knowledge acquired in Session 1; this is in contrast to the declarative paired associate task where we observed a sleep-associated benefit.

Participants then went on to complete two further blocks of category training; performance was assessed by comparing the number of correctly categorized trials across each block (see Table 4). An ANCOVA with the within-subject variable Block (Block 1, Block 2), between-subjects variable Group (15-min, PSG, 12-h wake, 12-h sleep) and covariate Test 1 revealed a main effect of Block, suggesting that participants were able to use the extra learning in session 2 to boost their category knowledge (F(1, 90) = 5.53, *p* = .021, η^2^ = 0.06). A main effect of Group was not observed (F(3, 90) = 1.88, *p* = .138) and there was no interaction with the factor Block (F(3, 90) = 2.61, *p* = .056, η^2^ = 0.08).

The third and final categorization test assessed category knowledge following both the consolidation delay and Session 2 training. Performance is shown in Fig. 5b. An ANCOVA with the factors Group (15-min, PSG, 12-h wake, 12-h sleep) and covariate Test 1 revealed a main effect of Group; F(3, 89) = 4.89, *p* = .003, η^2^ = 0.14. Bonferroni-corrected pairwise comparisons suggest that this main effect was driven by superior performance in the 12-h sleep (*p* = .009) and PSG (*p* = .008) groups in comparison to the 12-h wake condition. Participants who had sleep-filled consolidation delays, followed by further category training, showed higher rates of categorization compared to participants who stayed awake during the delay.

#### 3.3.3 Category learning – follow-up tasks

ANCOVAs with the variable Group (15-min, PSG, 12-h wake, 12-h sleep) and covariate Test 1 were performed separately for each follow-up task. Accuracy in the 2AFC and location task was calculated as the proportion of correct responses. Accuracy in the recall task was calculated as an error score, i.e. the difference between the participants response and the target response (the point of best fit based on the category distribution); a small error score is indicative of accurate performance in this task. All task scores are presented in Table 5; in the 2AFC and Location Task all groups performed above change level (chance = 0.5, *p*'s < .05). Group differences were not observed in the 2AFC task (F(3, 89) = 1.75, *p* = .163), the recall task (F(3, 89) = 2.25, *p* = .089) or the location task (F(3, 89) = 0.35, *p* = .788).

**Table 5:** Accuracy scores in the category follow-up tasks. Covariate adjusted means are presented (as evaluated with the covariate Test 1 accuracy). Standard error of the mean is presented in brackets.

	Categorization Follow-Up Tasks		
	2AFC (proportion correct)	Recall (error score)	Location Task (proportion correct)
15-min	0.62 (0.02)	39.17 (2.66)	0.57 (0.03)
PSG	0.61 (0.02)	37.95 (2.86)	0.57 (0.03)
12 h – Sleep	0.62 (0.02)	33.90 (2.92)	0.56 (0.03)
12 h – Wake	0.56 (0.02)	43.70 (2.84)	0.55 (0.03)

In Session 2 of this study participants completed multiple tests to assess the role of consolidation on the memory. Across these tests we find a significant effect of group in paired associate recall (*p* < .001) and in the third categorization task (*p* = .003). Given that we take multiple measures of performance across Session 2 (a total of 7 different measures) a more careful correction for multiple comparisons, including all post-consolidation tests, would be a Bonferroni corrected alpha level of *p* = .007 (.05/7). The significant effects of Group observed in this study survive this more conservative correction for multiple comparisons.

### 3.4 Sleep stage analysis

One participant was excluded from sleep analyses due to PSG equipment failure (N = 22). PSG recordings were scored in accordance with the criteria of the American Academy of Sleep Medicine (Iber et al., 2007). Sleep data was partitioned according to the proportion of total sleep time spent in stage I, stage II, slow-wave sleep (SWS) and rapid-eye-movement (REM) sleep. Sleep stage data is presented in Table 6. To establish whether the sleep related behavioural effects were driven by specific architectures of sleep, improvement scores were calculated between (i) delayed and immediate paired-associate recall, (ii) categorization accuracy in Test 2 and Test 1 and (iii) categorization accuracy in Test 3 and Test 1. Bivariate correlations were then performed between these behavioural measures and the proportion of time spent in (i) non-rapid-eye-movement (NREM) sleep (combined time in stage I, stage II and SWS), (ii) stage II sleep and (iii) and SWS were performed. Correlations for each behavioural measure were tested against a Bonferroni-corrected alpha level of *p ≤* .006.

**Table 6:** Percentage of time spent in each sleep stage. (NREM – non-rapid eye movement sleep, SWS – slow-wave sleep, REM – rapid eye movement sleep, TST – total sleep time). Standard error of the mean is presented in brackets.

NREM	Stage 1	Stage 2	SWS	REM	TST (min)
80.28 (0.74)	8.45 (0.66)	43.85 (1.32)	27.98 (1.43)	19.72 (0.74)	441.38 (11.10)

A positive correlation was observed between the proportion of time spent in NREM sleep and paired-associate learning (r = 0.514, *p* = .014) however this did not survive the Bonferroni corrected alpha level. Correlations with the proportion of time in stage II sleep (r = 0.317, *p* = .150) and SWS (r = 0.038, *p* = .868) were non-significant. No correlations were observed between improvement scores in the categorization task and each of the stages of sleep (all *p* > .5).

### 3.5 Model-based analyses

General Recognition Theory (GRT)-based analysis determines which of a predefined set of decision–boundary models best describes the classification adopted by each participant (Ashby and Gott, 1988). This analysis allows us to assess whether participants were truly adopting an information-integration decision boundary to separate Category A from Category B exemplars. Four models were considered in this analysis: one-dimensional, conjunction, general linear classifier and random.

The *one-dimensional* models assume that participants use a single dimension in order to classify stimuli by comparing each stimulus with a determined criterion value. An example using the tone frequency dimension in the current study would be “Respond Category A for high tones and Category B for low tones”. These models have two parameters: the criterion value and the variance of internal noise. The *conjunction* model suggests that participants hold a criterion value along both dimensions and combine the judgements to determine category membership. An example of a conjunction model would be “If the tone frequency is high and the pixel density is low assign Category A, otherwise assign Category B”. This model has three parameters: the two criterion values and internal noise. The *general linear classifier* (GLC) model assumes that a straight diagonal decision boundary can describe classification. The model can vary in gradient and intercept but suggests that participants are integrating across both dimensions to determine category membership. The GLC model has three parameters: the intercept, gradient and noise. The *random* model assumes that participants are responding randomly and this model has no parameters.

For each participant, and in each of the three categorization tests, the best fit of each of these models was calculated and the best fitting model was selected using Akaike's information criterion (Akaike, 1974). These analyses were performed using the grt package in R environment (Matsuki, 2017) and are reported in Table 7.

**Table 7:** Proportion of participants best described by each model according to the model-based analyses for each categorization test. (1D – one-dimensional, GLC – general linear classifier, CJ – conjunction, RND – random, T1 – Test 1, T2 – Test 2, T3 – Test 3).

	Strategies											
	1D			GLC			CJ			RND		
	T1	T2	T3	T1	T2	T3	T1	T2	T3	T1	T2	T3
15-minute	.48	.52	.31	.44	.30	.58	.04	.18	.08	.04	.00	.04
				
PSG	.48	.39	.22	.26	.39	.52	.22	.22	.22	.04	.00	.04
				
12-hour Sleep	.32	.41	.14	.50	.45	.77	.14	.09	.09	.05	.05	.00
				
12-hour Wake	.39	.39	.35	.61	.35	.39	.00	.26	.26	.00	.00	.00

**Fig. 6:**
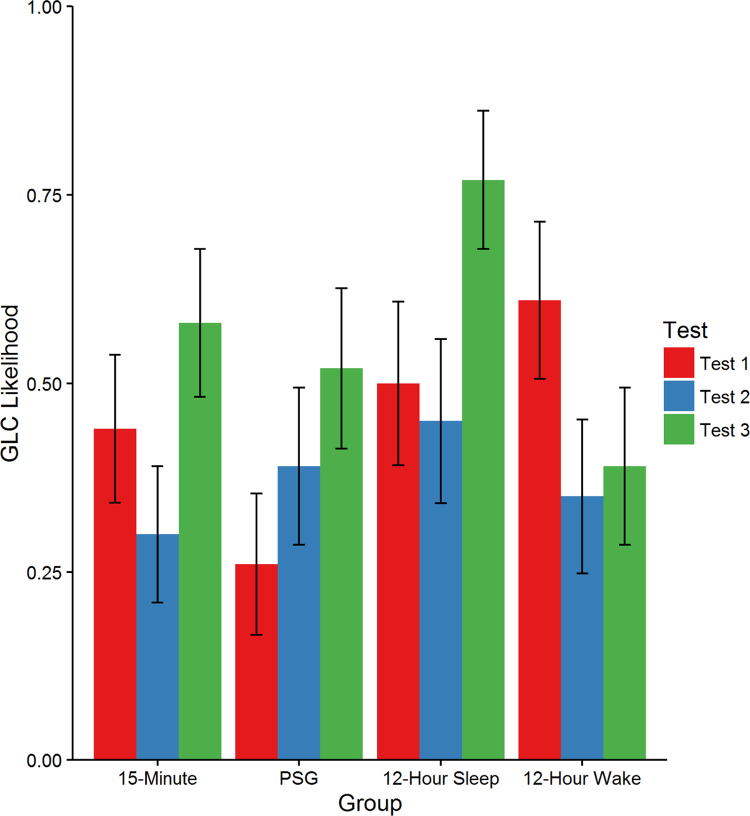
The likelihood of participants in each Group being classified as using the optimal GLC decisions boundary in the three categorization tests. Error bars represent standard error of the mean.

A mixed-effects model was fitted with the likelihood of a GLC classification as the dependent measure. The model included Group (15-min, PSG, 12-h sleep and 12-h wake), Test (Test 1, Test 2 and Test 3) and their interactions as fixed effects. Both fixed effects were coded with Helmert contrasts, with Test 1 and 15-min delay conditions acting as the reference levels. This meant that for Test a first contrast compared Test 1 with Tests 2 and 3, and a second contrast compared Test 2 with Test 3. For Group, a first test compared the three long delay groups (12-h wake, 12-h sleep and PSG) with the 15-min delay group, a second contrast compared the PSG and 12-h Sleep groups to the 12-h Wake group, and a third contrast compared the PSG and 12-h Sleep conditions. Random effects included by-subject intercepts only, which was the maximal random effect structure justified by the data (Baayen et al., 2008). We used the lme4 package in R with the logit link function (Bates et al., 2015, Jaeger, 2008) to conduct the analysis. There was a significant interaction between the second Group contrast (comparing the PSG and 12-h Sleep groups to the 12-h Wake group) and first Test contrast (comparing Test 1 with Tests 2 and 3), β = −0.24, standard error = 0.09, z = −2.83, *p* = .005. GLC classification in the PSG and 12-h sleep groups tended to increase between Test 1 and the two subsequent Tests, while there was a decrease in GLC classification in the 12-h Wake Group (see Fig. 6). There was also a significant effect for the second Test contrast (comparing Test 2 with Test 3), with all groups showing an increase in GLC classification across these two testing points (β = 0.53, standard error = 0.18, z = 2.95, *p* = .003). All other contrasts and interactions were non-significant (*p's* > .062).

Although modelling categorization data is typical in this area of research, the modelling results should be interpreted with caution given the restricted set of models tested and the small number of trials used for each test in the current study (Donkin et al., 2015).

## 4 Discussion

This study investigated the role of consolidation in both a declarative paired-associate memory task, and on the emergence of cross-modal conceptual representations using an information-integration categorization paradigm. In line with previous literature, we observed a clear sleep-associated consolidation benefit for paired-associate memory, with participants showing better retention following a consolidation delay that contained sleep compared to wakefulness. This result is consistent with the view that processes during sleep act to promote the consolidation of declarative memory (Diekelmann et al., 2009, Rasch and Born, 2013). Our assessments of category knowledge provide good evidence for sensory-integration, with participants successfully acquiring the cross-modal (auditory – visual) category structure. As real-world conceptual knowledge comprises information across multiple modality dimensions (Patterson et al., 2007) this task, albeit in a very simplistic form, resonates with natural concept learning. However, in contrast to paired-associate memory, we did not observe any immediate post-delay wake- or sleep-associated changes in categorization accuracy. Instead, we found a facilitative effect of sleep-associated consolidation on subsequent learning, with participants showing greater category knowledge and shifts towards more optimal decision strategies after training in session two, if they had a delay filled with sleep.

These results suggest that the behavioural benefits of sleep-associated consolidation are dependent upon the type of memory being assessed. Episodic memory, as assessed by the paired-associate task, produces immediate sleep benefits in memory recall, whereas the advantages for conceptual memory emerge only after an opportunity for further learning. This result draws attention to the relationship between sleep-associated consolidation and the effectiveness of post-consolidation learning; an important finding when considering the development of conceptual memory which develops across temporally distinct episodes interleaved with consolidation opportunities.

These results are in agreement with theories of consolidation which suggest that sleep facilitates systems-level memory reorganisation, allowing new and consistent information to be assimilated into long-term memory networks at a quicker rate (McClelland, 2013, McClelland et al., 1995; Kumaran et al., 2016; Tse et al., 2007; van Kesteren et al., 2013). Sleep-dependent training benefits in this study may therefore be the consequence of subtle sleep-dependent mechanisms which facilitated the storage of category knowledge acquired in session one; thus providing the architecture required for enhanced assimilation of new and consistent information the following day. This interpretation is also supported by modelling the decision strategies of participants; those who had the opportunity to sleep between sessions showed a shift to the optimal linear decision strategy following the delay and session two training. Memory reorganisation during sleep, which may promote the development of category structure, along with further task training, may have allowed participants to align their response strategies with the optimal linear decision boundary in this task. This same shift in response strategy was not observed following 12-h of wakefulness, supporting the suggestion of a sleep-associated mechanism in the consolidation of category knowledge.

These results highlight the importance of assessing consolidation across multiple learning episodes when studying the development of categorical memory representations. An interesting question that remains is whether the benefits of sleep on second session learning are specific to the trained categorization structure, or whether these benefits extend to perceptually and/or structurally similar categorization tasks. Understanding the flexibility of consolidated categorical representations will be important for determining the role of consolidation in broader conceptual memory.

We observed differences in the sleep-associated benefit observed across the two tasks in this study. One possible reason for this is due to the nature of encoding. Paired-associate learning requires participants to make associations between two previously unrelated items, creating very strong episodic memory representations which place high demands on the medial temporal lobe system in the brain, in particular the hippocampus (Cameron et al., 2001). The hippocampus plays a pivotal role in theories of memory consolidation, with the suggestion that it is responsible for both the rapid encoding of information during wake and then the redistribution of encoded material to the neocortex during sleep (McClelland et al., 1995, Diekelmann and Born, 2010). In contrast to paired-associate learning, the categorization task considerably reduces the value of episodic encoding by using a continuous category structure without a definitive category boundary (i.e. there was a degree of category overlap). This results in each trial being perceptually very similar, without any discriminative or arbitrary features to allow trial-by-trial individuation

The immediate sleep-dependent benefit for paired-associates may therefore reflect a component of the consolidation mechanism which is strongly linked to episodic memory. We were not able to compare episodic and conceptual memory within the same paradigm in the current study, however Graveline and Wamsley (2017) were able to do this using a classification task in which participants were trained to discriminate between dot patterns that were derived from category prototypes. Importantly, participants were trained on individual category exemplars, that although perceptually very similar, were repeatedly presented during training, allowing participants to develop strong representations for individual items. In line with our paired-associate data, they show sleep-dependent benefits in memory for these trained items. However, they also show sleep benefits for the categorization of novel and untrained category patterns, suggesting that sleep also benefitted the extraction of shared category knowledge. This highlights a complex interplay between episodic and conceptual memory, where sleep may benefit concept based representations when strong individual episodic representation are held in memory.

The sleep-dependent benefit in post-consolidation learning in this study is in contrast to the wake-dependent consolidation benefit observed in the category learning study by Hennies et al. (2014). In a similar categorization task they found that wake, rather than sleep, facilitated the development of category knowledge. Two factors may account for these contradictory results; the first is the selectivity of sleep-dependent consolidation (Rasch and Born, 2013). Sleep-dependent consolidation effects are more robust under explicit learning conditions and are improved by motivational factors such as relevance for future goals (Robertson et al., 2004, Fischer et al., 2006, Walker et al., 2003, Cohen et al., 2005, Diekelmann et al., 2008, Wilhelm et al., 2011). In the current study, participants were explicitly aware of the relevant information needed for determining category membership (i.e. the visual and auditory dimensions) despite the nature of the category structure itself being initially unknown. In contrast, the underlying category structure was truly implicit in Hennies et al. (2014). They manipulated the traditional categorization paradigm such that the information-integration category structure was hidden within a pre-stimulus event, which if utilised would increase reaction time, but was not necessary for accurate categorization. Explicit appreciation for the relevant integrative dimensions may therefore make the stimulus in this experiment more susceptible to sleep-dependent consolidation mechanisms.

A second factor that may explain the differences observed between these studies relates to the level of initial learning. Stickgold (2009) proposed that sleep mainly benefits memories encoded at intermediate memory strengths, such that there is an inverted-U shaped curve to the sleep benefit. As a result, both very weak and very strong memories would fail to benefit from sleep-based consolidation mechanisms. In the current study participants were able to categorize stimuli above chance level after training in session one, but did not reach ceiling levels. According to the theory proposed by Stickgold (2009), learning was therefore within the optimal range to benefit from sleep-dependent consolidation. In contrast, Hennies et al. (2014) found no evidence of implicit category learning before the consolidation delay; participants may have been insensitive to sleep-dependent consolidation mechanisms in their study.

Given that the results of the current study contrast with those from Hennies et al. (2014) it is important to note that we did provide a direct replication of our sleep effect by using two sleep group comparisons. This study was initially run as a comparison between two groups with a 12-h delay containing wake or sleep. Following data collection and preliminary analyses, the 15-min and PSG monitored group were added to i) provide a short delay comparison and ii) to replicate the sleep effect observed in the initial 12-h sleep group with concurrent PSG recordings. We successfully replicated the initial sleep-associated benefit but present all groups within a single comparison in the current paper to streamline the analysis. Replication of the sleep benefit observed in this study, as well as further investigation more generally within the domain of consolidation and categorization is certainly required to fully understand the development of category knowledge across time. The design we used in this experiment, which compares nocturnal sleep with daytime wakefulness, like many others is the consolidation literature, does not control for circadian effects on memory that may influence performance (Rasch and Born, 2013). Although ratings of sleepiness and vigilance suggest that participants' general alertness levels were comparable in the current study, a replication of the sleep-based effects using a nap design would remove this confound and add support to our interpretations.

This study compared the role of consolidation in a declarative paired-associate task, and on the emergence of cross-modal categorical memory representations. We provide good evidence for a role of sleep-dependent consolidation in paired-associate learning, with participants showing post-sleep benefits in memory recall that correlate with signatures of sleep. This finding is in line with a growing body of research suggesting that processes during sleep play an active role in the consolidation of declarative memory (Rasch and Born, 2013). Using a perceptual categorization task, we were able to demonstrate cross-modal category learning, a key feature of real-world conceptual memory for which information is drawn from multiple sensory dimensions. We also observe a sleep-dependent consolidation benefit in category learning; however unlike paired-associate memory, this benefit emerges only when sleep-based consolidation is paired with further category training. This result highlights an important interaction between those mechanisms responsible for consolidation and those responsible for learning. Establishing the exact nature of this relationship will be important for (i) understanding how we develop, update and maintain conceptual memory representations and (ii) understanding why we observe different behavioural consequences of sleep-dependent consolidation across episodic declarative and conceptual memory representations.
